# WNT16 Regulation of the Articular Chondrocyte Phenotype in Mice

**DOI:** 10.3390/life13040878

**Published:** 2023-03-25

**Authors:** Subburaman Mohan, Shelia Pourteymoor, Chandrasekhar Kesavan

**Affiliations:** 1 Musculoskeletal Disease Center, VA Loma Linda Healthcare System, Loma Linda, CA 92357, USA; 2 Department of Medicine, Loma Linda University, Loma Linda, CA 92354, USA; 3 Orthopedic Surgery, Loma Linda University, Loma Linda, CA 92354, USA

**Keywords:** WNT16, osteoarthritis, chondrocytes, mice, chondrogenesis, cell culture

## Abstract

The anabolic effects of WNT16 on osteoblasts are well established, however, little is known regarding the role of WNT16 in chondrocytes. In this study, we evaluated *Wnt16* expression and its biological effects on mouse articular chondrocytes (ACs), since these cells are key to the development of osteoarthritis. While ACs derived from the long bone epiphysis of 7-day old C57BL/6J mice express multiple *Wnt*s, *Wnt5b and Wnt16* represent the two most highly expressed *Wnt*s (expressed at several-fold higher levels than other *Wnt*s). Treatment of serum-free AC cultures, with 100 ng/mL of recombinant human (rh) WNT16 for 24 h (hrs), increased proliferation (20%, *p* < 0.05) and expression levels of makers (*Sox9 and Col2*) of immature chondrocytes at both 24 h and 72 h, while *Acan* increased at 72 h. Expression of *Mmp9*, a marker of mature chondrocytes was decreased at 24 h. Additionally, WNT16 treatment regulated expression levels of *Wnt* ligands in a biphasic manner, inhibiting its expression at 24 h, while stimulating expression at 72 h. To determine whether WNT16 exerted anabolic effects on the AC phenotype, ex vivo cultures of tibial epiphyses were treated with rhWNT16 or vehicle for 9 days, and the articular cartilage phenotype was evaluated by safranin O cartilage staining and expression of articular cartilage marker genes. Both articular cartilage area and expression levels of AC markers were increased after rhWNT16 treatment. Our data suggest that Wnt16 expressed in ACs may play a role in regulating joint cartilage homeostasis via its direct effect, as well as through modulating the expression of other *Wnt* ligands.

## 1. Introduction

The cartilage around the joints acts as a cushion between bones in healthy individuals, by absorbing shock during physical activity. In response to acute joint trauma, obesity, aging and bone fracture, cartilage tends to deteriorate because of the activation of sequential events, including inflammation, apoptosis and matrix degradation, which triggers a chronic remodeling process in the joint cartilage over time [1,2]. This remodeling leads to a clinical condition known as osteoarthritis (OA), a progressive degenerative joint cartilage disease affecting over 240 million people globally (10% men and 18% women), especially in our aging population, leading to disability, a poor quality of life and an increased mortality rate [3,4,5]. Presently, there are no available effective therapies for OA [6,7]. Therefore, understanding the biology of articular cartilage development and identifying signaling mechanisms that are perturbed during OA is important for developing future strategies to treat joint disease.

In terms of regulatory molecules important for articular cartilage development, recent clinical studies have reported increased Wingless-related Integration site (*Wnt*) *16* expression in injured joints, in the temporomandibular junction after blocking canonical WNT signaling, and in lumbar facet joint OA. However, it was unclear if this increased expression initiated anabolic or catabolic effects [8,9,10,11,12]. In response to injury, transgenic animal studies have reported that mice deficient in *Wnt16* expression exhibited a severe OA phenotype compared with wild-type mice. [13]. While these studies have implicated a role for *Wnt16* in the articular cartilage phenotype, the issue of whether *Wnt16* is expressed in articular chondrocytes and exerts significant biological effects on articular chondrocytes remains to be established. The findings of this study demonstrate that articular chondrocytes expressed *Wnt16*, and that exogenous treatment with recombinant WNT16 promoted the articular chondrocyte phenotype, as evidenced by increased chondrocyte proliferation and expression of articular cartilage marker genes.

## 2. Materials and Methods

### 2.1. Chondrocyte Culture

Cells from the epiphyseal region of long bones were collected from euthanized 7-day old C57BL/6J mice (*n* = 6), as described [14]. The cells were cultured in alpha-minimal essential medium (αMEM) containing 10% fetal bovine serum (FBS) with antibiotics (penicillin 100 units/mL and streptomycin 100 µg/mL), for 3 days. The cells were passaged once, and the first-passage cells were used for experiments.

### 2.2. Gene Expression

Approximately 200,000 cells were platted per well in 6-well plates, and cultured in αMEM containing 10% FBS with antibiotics for 48 h (hrs), followed by another 24 h in αMEM containing 0.1% bone serum albumin (BSA) and antibiotics. Thereafter, the cells were treated with or without 100 ng/mL of recombinant human (rh) WNT16 (R&D Systems, Minneapolis, MN, USA) or vehicle (1X phosphate buffered saline), and were cultured in αMEM containing 0.1% BSA and antibiotics. The experiments were terminated 24 or 72 h after WNT16 treatment. Total ribonucleic acid (RNA) was isolated from cells treated with vehicle and WNT16 using a Qiagen isolation kit protocol, per the manufacturer’s instructions. Two hundred nanograms of purified total RNA was used to synthesize the first strand complementary deoxyribose nuclei acid (cDNA) by reverse transcription, as per the manufacturer’s instructions (Bio-Rad, Irvine, CA, USA). The first strand of DNA was subjected to real-time PCR amplification, using a SYBR green master mix and gene-specific primers (IDT DNA Technology, San Diego, CA, USA) on a ViiA7 real-time PCR system (Applied Biosystems, Waltham, MA, USA). The endogenous control (*18S*) was used to normalize the data [15,16], and the normalized values were subjected to the 2^∆∆^Ct (where C+ is contraction threshold) formula, to calculate the fold change between the vehicle and experimental groups [17].

### 2.3. Proliferation Assay

The first-passage chondrocytes (4000 cells/well) were plated in a 96-well plate and cultured in αMEM containing 10% FBS with antibiotics for 48 h, followed by another 24 h in αMEM containing 0.1% BSA with antibiotics. The cells were then treated with vehicle (1X PBS) or 100 ng/mL of rhWNT16 (R&D System, Minneapolis, MN, USA) in αMEM containing 0.1% BSA and antibiotics. Proliferation was assessed 48 h after WNT16 treatment using a Cy-Quant Dye kit (Life Technologies, Carlsbad, CA, USA), according to the manufacturer’s instructions.

### 2.4. Ex Vivo Joint Culture and Histology

We collected right and left intact femoral head bones that were connected to the hip, from euthanized 6-month-old male C57BL/6J mice. Briefly, the whole intact femoral head was isolated by cutting at the junction of the femoral neck. The femoral head width was approximately 0.95–1 mm, and the length from the head to the neck junction was 1 mm. The femoral head was cultured in αMEM with 0.1% BSA and antibiotics in 96-well plates. A 100 µL medium was added to each well so that the femoral head was completely immersed in the media. The media was replaced every day. The right femoral head joint was treated with 100 ng of rhWNT16 once/day for 9 days, while the corresponding left femoral head joint was treated with vehicle (1X PBS). Nine days after WNT16 or vehicle treatment, the femoral heads were fixed in 10% formalin for 24 h, and then washed and stored in 1X PBS. Six-micron paraffin-embedded sections were prepared from these samples, processed, and stained with safranin O [14]. The stained area was outlined in both the rhWNT16- and vehicle-treated groups, using the Image J software. The difference in the staining area was calculated as a percentage using this formula: (treated value − vehicle value)/vehicle value ∗ 100).

### 2.5. Ex Vivo Joint Culture and Gene Expression

Right and left proximal tibial epiphyses, with attached articular cartilage, were collected from euthanized 6-month and 10-month-old male C57BL/6J mice under sterile conditions, and cultured in αMEM with 0.1% BSA and antibiotics. The right tibial articular cartilage was treated with 100 ng/mL of rhWNT16 once/day for 7 days, while the corresponding left tibia articular cartilage was treated with vehicle (1X PBS). The media was changed every day for both groups. Seven days after WNT16 treatment, the tibia epiphyses with articular cartilage from 6-month and 10-month-old mice were used for total RNA isolation (Qiagen, MD, USA). Purified total RNA was used to synthesize the first strand of cDNA by reverse transcription, according to the manufacturer’s instructions (Bio-Rad, CA, USA). Quantitative real-time PCR was used to determine the expression levels of genes using the SYBR green dye approach, as described above. Gene-specific primers were designed with the Vector NTI software and ordered from IDT DNA technologies. Data normalization was accomplished using an endogenous control (*18s*) to correct for variations in the RNA quality among samples. The normalized CT values were used to calculate the fold change using the 2^−∆∆^Ct formula [17].

### 2.6. Statistical Analysis

The Student *t*-test was used to compare the difference between the treatment vs. non-treatment groups. A *p*-value of <0.05 was considered statistically significant. Values are presented as the mean ± standard error mean (SEM).

## 3. Results and Discussion

**Figure 1:**
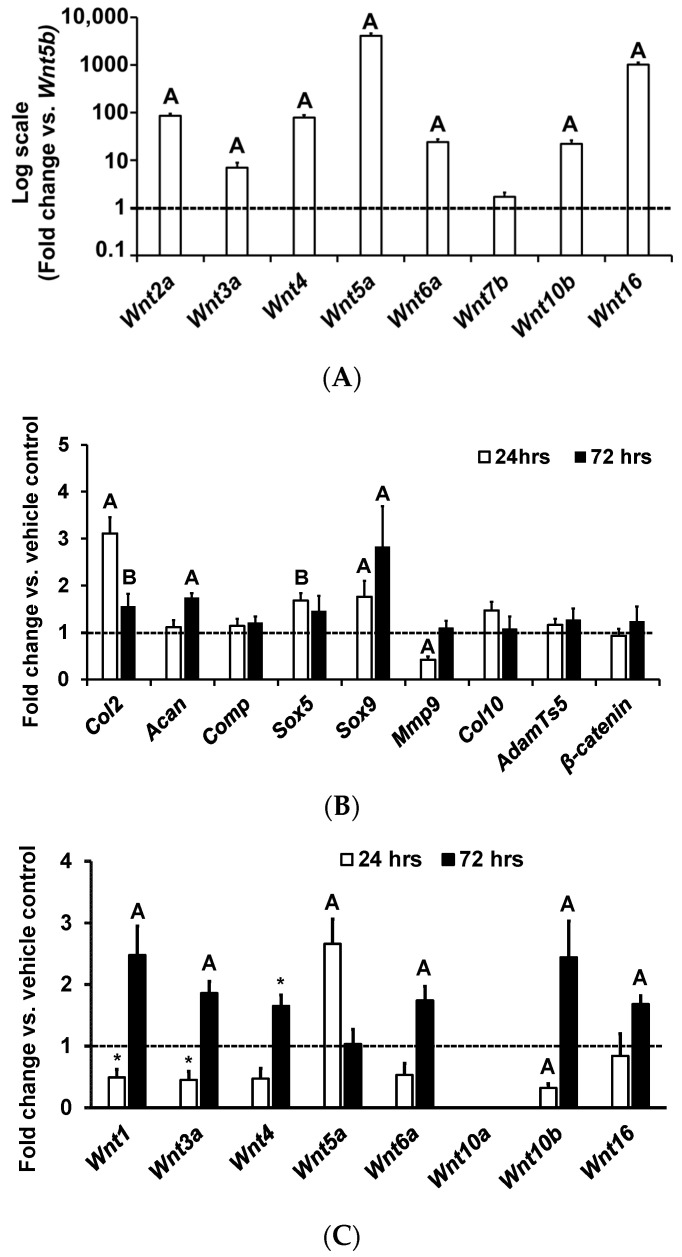
Expression patterns of *Wnt*s and chondrocyte marker genes in primary articular chondrocyte cultures in vitro. (**A**) Expression of *Wnt*s in cultured articular chondrocytes isolated from 1-week-old C57BL/6J mice (fold-change data vs. *Wnt5b* is expressed in the log scale). (**B**) Quantitation of expression of genes 24 (*n* = 5) and 72 h post rhWNT*16* treatment (*n* = 5) in chondrocytes derived from epiphyses of 1-week-old C57BL/6J mice. (**C**) Quantitation of expression of *Wnt* ligands 24 and 72 h post rhWNT16 treatment in chondrocytes isolated from the epiphyses of 1-week-old C57BL/6J mice (*n* = 3). Values are the mean ± SEM. ^A^ *p* < 0.05 and ^B^ *p* = 0.06 vs. vehicle, * *p* = 0.08 vs. vehicle. The *x*-axis represents the genes studied and *y*-axis reflects fold-change in expression.

Clinical and animal model studies have reported increased *Wnt16* expression in OA knees compared to control knees. Furthermore, it has been shown that Wnt16 treatment of human cartilage explants via a peptide-based nanoplatform to deliver *Wnt16* mRNA was effective in maintaining cartilage homeostasis, and that over-expression of *Wnt16* using an adenovirus reduced the progression of OA in an animal model [18,19]. While these findings suggest a role for WNT16 in the pathogenesis of OA, the issue of whether *Wnt16* is expressed in articular chondrocytes and exerts significant biological effects remains to be examined. We, therefore, evaluated the expression levels of *Wnt* ligands by real-time PCR in the RNA samples isolated from articular chondrocytes of 7-day old C57BL/J mice. We used the *18s* gene as an endogenous control to normalize our expression data. Of the *Wnt*s examined, *Wnt5b* showed the lowest expression, and was therefore used as a baseline to compare the expression of the other *Wnt* ligands. We found that, except for *Wnt10a* (below detectable limit), all other *Wnt*s examined were expressed in articular chondrocytes, but at different levels (Figure 1A). *Wnt5a* and *Wnt16* were found to be expressed at much higher levels than other *Wnt*s in articular chondrocytes. Both canonical (*Wnt*s *2a, 3a* and *10b*) and non-canonical (*Wnt*s *4, 5a, 6a and 16*) *Wnt*s were expressed by articular chondrocytes, suggesting that both signaling pathways may be involved in the development and maintenance of the articular cartilage phenotype.

To determine the WNT16 biological effects on chondrocytes, we tested if rhWNT16 modulates proliferation of articular chondrocytes. We chose a 100 ng/mL dosage based on a dose-response study that revealed a maximum biological effect at this dose in MC3T3-E1 mouse osteoblasts [20]. Furthermore, we chose human rhWNT16 protein for the study, because the WNT16 protein is 92% conserved between mouse and human, and the long-term goal of research is to determine if we could use human rhWNT16 protein as a therapy to promote joint cartilage homeostasis. We found that cells treated with rhWNT16 showed a 20% increase in proliferation 48 h after WNT16 treatment. Terminal differentiation of articular chondrocytes into hypertrophic chondrocytes is a known cause for the pathogenesis of osteoarthritis [21]. We, therefore, determined the effects of WNT16 on expression levels of markers of the transition of immature to mature chondrocytes [22]. We found that the markers that reflect the transition of stem cells to chondrocytes *Sox*5 (*p* = 0.06), *Sox9* (*p* = 0.04) and type-2 collagen (*Col2*) (*p* < 0.01)) (Figure 1B) increased in response to WNT16 treatment. By contrast, a marker that is highly expressed in hypertrophic chondrocytes that undergoes apoptosis, *Mmp9*, was reduced (*p* < 0.01) 24 h post WNT16 treatment. After 72 h treatment with rhWNT16, expression levels of the articular cartilage markers, *Sox5* (*p* < 0.05), *Col2* (*p* < 0.05) and aggrecan (*Acan* (*p* = 0.06)) remained increased, showing a sustained anabolic effect (Figure 1B). Importantly, the decrease in *Mmp9* and increase in expression of transcription factors (*Sox5, 9*) upon WNT16 treatment suggests that the WNT16 treatment acts to maintain the articular chondrocyte fate. In our study, we chose a serum-free medium (0.1% BSA) to test the effects of exogenously added Wnt16, since sera contain a number of growth factors that could interact with Wnt16 effects and make the data difficult to interpret. In future studies, we will determine if serum growth factors modify Wnt16 biological effects in chondrocytes.

To determine if the WNT16 effects were mediated in part by modulating the expression of other Wnt ligands, we evaluated expression levels of *Wnt*s known to influence chondrogenesis 24 or 72 h after rhWNT16 treatment in articular chondrocytes (Figure 1C). We found that WNT16 affects the expression of multiple *Wnt*s in articular chondrocytes. Twenty-four-hour WNT16 treatment suppressed the expression of *Wnt1*, *Wnt3a* and *Wnt10b*, and increased the expression of *Wnt5a*, which has been reported to have an anabolic effect on cartilage (Figure 1C). However, at 72 h WNT16 treatment increased expression of *Wnt1*, *Wnt3a, Wnt4, Wnt6A, Wnt10b and Wnt16*. Overall, the expression data suggest that WNT16 modulates expression of other *Wnt*s, in both a positive and negative manner.

**Figure 2:**
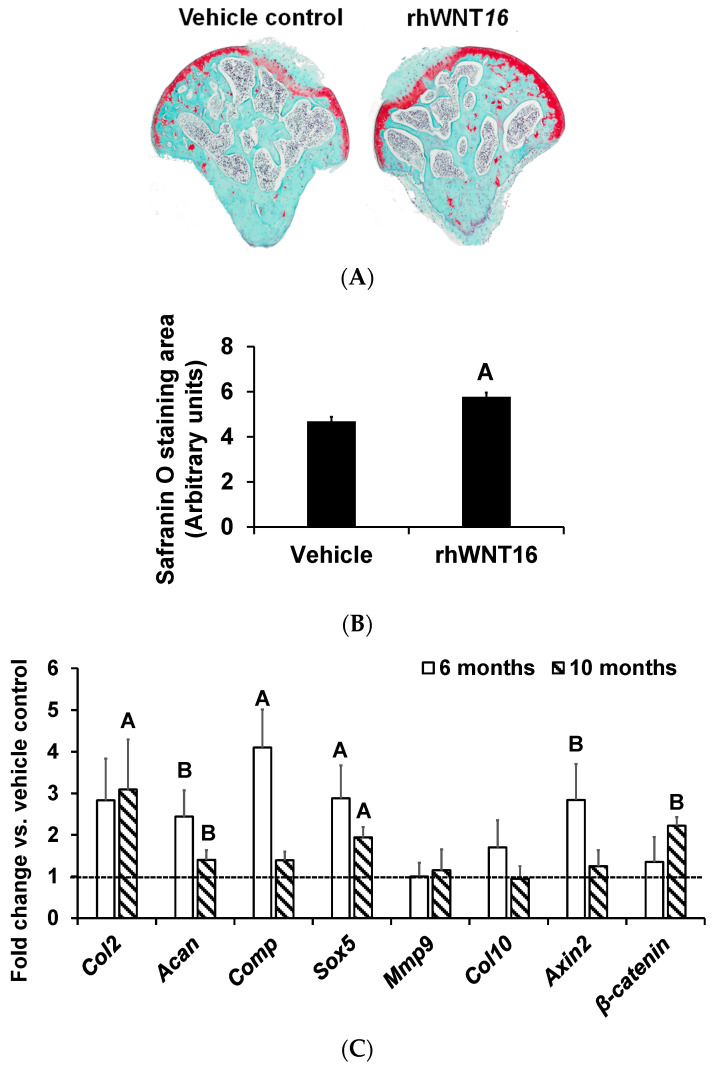
Illustration of the femoral head safranin O staining and expression patterns of chondrocyte marker genes in ex vivo cultures of tibia epiphysis regions which contain articular cartilage. (**A**) Histology image of safranin O stained areas and (**B**) quantitation of safranin-O-stained areas in 6-month-old male femoral heads cultured ex vivo and treated with rhWNT16 vs. vehicle. (**C**) Quantitation of expression levels of genes from epiphyseal regions of the tibia with articular cartilage, isolated from 6- and 10-month-old male C57BL/6J mice and cultured ex vivo with 100 ng rhWNT16 vs. vehicle for 7 days in vitro. *n* = 5/group, values are the mean ± SEM, ^A^ *p* < 0.05 vs. vehicle and ^B^ *p* = 0.08 vs. vehicle.

In humans, joint cartilage damage is very common in young adults and middle-aged adults. To determine if WNT16 could produce anabolic effects in the articular cartilage of adult mice, we isolated the femoral head up to the neck junction with intact articular surfaces from 6-month-old male (corresponds to adult humans) C57BL/6J mice, and cultured them for 9 days with and without rhWNT16. Staining of the histological femoral head sections with safranin O revealed an increased articular cartilage staining area in femoral heads treated with WNT16, compared to joints treated with the vehicle (Figure 2A,B). Consistent with the histology data showing the anabolic effects of rhWNT16, we found that a 7-day treatment with rhWNT16 increased the expression levels of markers of chondrogenesis (*Col2, Sox5, Acan, β-catenin* and *Comp*) in cultured tibia epiphyseal articular cartilage regions from 6-month-old, as well as from 10-month-old (corresponds to middle-aged humans) C57BL/6J mice, compared to the vehicle-treated cultures (Figure 2C).

The limitations of this study include a lack of confirmation of gene expression data by corresponding changes in protein levels, and the failure to establish a causal role for Wnt16 expressed in articular chondrocytes in regulating the articular cartilage phenotype. Our future investigations will focus on the in vivo role of Wnt16 in regulating the articular cartilage phenotype.

## 4. Conclusions

Articular chondrocytes express WNT16, and exogenous treatment with WNT16 stimulates mRNA levels of the genes involved in regulating joint cartilage homeostasis directly, as well as through modulating expression of other *Wnt* ligands.

## Data Availability

Raw data are available upon request.
